# FH535-mediated inhibition of Wnt/β-catenin suppresses CRC growth and invasion and alters MCP1 and MCP2 expression with potential immunological implications

**DOI:** 10.3389/fonc.2026.1800308

**Published:** 2026-04-01

**Authors:** Aisha Saleh Janeeh, Bilal Rah, Jasmin Shafarin, Khuloud Bajbouj, Eman Abu-Gharbieh, Mawieh Hamad

**Affiliations:** 1 Iron Biology Research Group, Research Institute for Medical Research, University of Sharjah, Sharjah, United Arab Emirates; 2 Department of Clinical Sciences, College of Medicine, University of Sharjah, Sharjah, United Arab Emirates; 3 Department of Biomedical Sciences, School of Veterinary Medicine, University of Pennsylvania, Philadelphia, PA, United States; 4 Biopharmaceutics and Clinical Pharmacy, Faculty of Pharmacy, The University of Jordan, Amman, Jordan; 5 Department of Medical Laboratory Sciences, College of Health Sciences, University of Sharjah, Sharjah, United Arab Emirates

**Keywords:** colorectal cancer, FH535, monocyte chemoattractant proteins, tumor microenvironment, Wnt/β-catenin pathway

## Abstract

**Background:**

The Wnt/β-catenin signaling pathway plays a key role in colorectal cancer (CRC) progression, but its broader biological effects, when blocked, remain poorly understood. In this study, we examined the direct antitumor effects and the impact on the tumor microenvironment of FH535, a small-molecule inhibitor of Wnt/β-catenin and PPARδ signaling, in human CRC cells.

**Methods:**

We treated HCT116 and HT29 colorectal cancer cell lines with different doses of FH535. We measured changes in cell viability, colony-forming ability, movement, invasion, cell death, and cell-cycle stages. We used Western blotting to check key proteins involved in Wnt/β-catenin signaling, cell-cycle control, DNA damage, and cell death. To make sure the effects were specific to the pathway, we also used siRNA to silence β-catenin. We then measured the levels of the chemokines MCP1 (CCL2) and MCP2 (CCL8) after both drug treatment and gene silencing.

**Results:**

FH535 treatment reduced cell viability and colony-forming ability as the dose increased. It also reduced the cells’ ability to move and invade. These changes were accompanied by increased cell death, as evidenced by flow cytometry and caspase-3 cleavage. Levels of total and phosphorylated β-catenin, cyclin D, and survivin also went down. Both FH535 treatment and β-catenin silencing strongly reduced MCP1 and MCP2 levels.

**Conclusion:**

FH535 shows antitumor effects in colorectal cancer by blocking Wnt/β-catenin signaling. This results in reduced cell growth, decreased movement, and increased cell death. Lower levels of MCP1 and MCP2 suggest this pathway also affects immune responses. These results support targeting Wnt/β-catenin as a possible treatment for CRC.

## 1 Introduction

CRC is the third most prevalent malignancy, contributing to approximately 10% of diagnosed cancers and nearly 6% of cancer-related mortality worldwide. In 2021 alone, over 1.9 million new CRC cases were reported globally, underscoring its significant public health burden (1). CRC development is a complex, multistep process that can take 10 to 20 years to be diagnosed using standard methods. CRC development and progression are driven by a sequential accumulation of inter- or intra-tumoral genetic and epigenetic changes that disrupt normal colonic epithelial homeostasis (2). Several molecular pathways contribute to CRC pathogenesis, including microsatellite instability (MSI), chromosomal instability (CIN), and CpG island methylator phenotype (CIMP), reflecting CRC’s molecular heterogeneity and complexity (3, 4). Mutations in the tumor suppressor gene *adenomatous polyposis coli* (APC) represent a critical early event that dysregulates the Wnt/β-catenin signaling cascade, a key driver of CRC initiation and progression (5).

The Wnt signaling pathway, first elucidated through the discovery of the *wingless* (*wg*) gene in *Drosophila melanogaster*, orchestrates essential cellular processes such as proliferation, polarity, differentiation, stemness, and migration during embryogenesis and adult tissue homeostasis (6, 7). Canonical Wnt signaling involves β-catenin stabilization and nuclear translocation, regulating the transcription of genes implicated in cell cycle progression and survival. In contrast, non-canonical Wnt pathways govern planar cell polarity and intracellular calcium dynamics (8). Dysregulated canonical Wnt signaling in CRC fosters uncontrolled proliferation, epithelial–mesenchymal transition (EMT), and resistance to apoptosis.

Beyond intrinsic oncogenic signaling, CRC progression is profoundly influenced by the tumor microenvironment (TME), particularly tumor-associated macrophages (TAMs). TAMs, recruited by cancer-derived chemokines, actively support tumor growth, immune evasion, angiogenesis, and metastasis (9, 10). Among these chemokines, monocyte chemoattractant proteins, including MCP1 (CCL2) and MCP2 (CCL8), have emerged as key mediators of macrophage infiltration and polarization within the TME (11). These MCPs can promote EMT, thereby enhancing migratory and invasive capacities (12–14). Targeting the interplay between oncogenic pathways, such as the Wnt/β-catenin pathway, and chemokine-mediated modulation of the TME represents a promising dual therapeutic strategy in CRC. FH535 is a bifunctional small-molecule antagonist targeting both peroxisome proliferator-activated receptors (PPARs) and the β-catenin/TCF/LEF signaling cascade (15). Previous studies have demonstrated that FH535 effectively suppresses the proliferation of various malignancies, including CRC, lung, breast, and pancreatic cancers, as well as hepatocellular carcinoma (HCC) cell lines (16–18). Furthermore, it was reported to attenuate pancreatic tumor xenograft progression and inhibit tumor-associated angiogenesis (19). However, the effect of Wnt/β-catenin inhibition on MCP expression in CRC remains unknown.

Although pharmacologic inhibition of Wnt/β-catenin signaling, including FH535, has demonstrated anti-proliferative effects in colorectal cancer (CRC), the downstream inflammatory and chemokine networks regulated by this pathway have received less attention. Precision oncology now considers not only pathway dependency but also context-specific downstream effects that shape tumor–immune interactions and therapeutic response. Chemokines such as MCP1/CCL2 and MCP2/CCL8 play key roles in immune cell recruitment, modulation of the tumor microenvironment, and disease progression. The mechanisms by which β-catenin signaling regulates MCP1/CCL2 or MCP2/CCL8 in CRC remain unclear.

This study examines whether inhibiting β-catenin signaling alters MCP1/CCL2 and MCP2/CCL8 expression in CRC cells, with the goal of clarifying the connection between canonical Wnt signaling and chemokine-mediated tumor–immune interactions. Herein, we addressed this issue *in vitro* by assessing MCP1 and MCP2 expression in FH535-treated or Wnt/β-catenin-silenced CRC cells (HCT116 and/or HT29). The impact of Wnt/β-catenin inhibition or depletion on CRC cell viability, apoptosis, migration, and invasion was also evaluated.

## 2 Materials and methods

### 2.1 Cell line preparation

Human CRC cell lines HCT116 and HT29 were obtained from Research Institute for Medical and Health Sciences (RIMHS), University of Sharjah, UAE, and cultured in a 75 cm^2^ petri dish with 10 mL of Rose Well Park Memorial Institute-1640 (RPMI-1640) medium (RNBH1005, Sigma-Aldrich) supplemented with 10% heat‐inactivated fetal bovine serum (FBS) (Sigma-Aldrich) and 1% penicillin‐streptomycin (Sigma-Aldrich) at 37 °C in a humidified atmosphere containing 5% CO_2_. Human monocytic THP-1 cells were cultured in RPMI 1640 medium supplemented with 10% heat-inactivated fetal bovine serum and 1% antibiotic solution. The media was refreshed 2–3 times per week to maintain optimal growth conditions. The cells were passaged when they reached approximately 80% to 90% confluence.

### 2.2 Treatment protocols

Cells were treated with a Wnt antagonist, FH535 (SC-221398, Santa Cruz Biotechnology), at the 24-hour time point, and a β-catenin/Tcf inhibitor that suppresses canonical Wnt signaling and its downstream mediators.

### 2.3 MTT (3-(4, 5-dimethylthiazolyl-2)-2, 5-diphenyltetrazolium bromide) assay

A total of 5 × 10^3^ CRC (HCT116 and HT29) cells/well were seeded in 96-well plates in triplicate. The following day, cells were treated with varying concentrations of Wnt antagonist (10, 20, 30, 40, 50, 60, and 70 μM). Treated plates were incubated for 24 hours at 37 °C in a humidified atmosphere containing 5% CO_2_ to allow cells to reach approximately 80% confluency. After incubation, 10 µL of MTT reagent (M5655, Sigma-Aldrich) was added to each well, and the plates were incubated for an additional 2 h. Subsequently, the media was removed, and 100 µL of dimethyl sulfoxide (DMSO) was added to dissolve the formazan crystals. Absorbance was measured using a microplate reader at 570 nm.

### 2.4 Phase contrast microscopy

For phase-contrast microscopy, CRC (HCT116 and HT29) cells (0.5 × 10^6^) were seeded into 6-well plates and incubated overnight to allow proper adhesion to the culture surface. The following day, cells were treated with varying concentrations (3.75, 7.5, 15, 30, and 60 µM) of the FH535 antagonist, while an untreated group served as the control. After 24 hours of treatment, cellular morphology was examined under a phase-contrast microscope at (10×) magnification to assess morphological alterations in response to FH535 exposure relative to untreated controls.

### 2.5 Crystal violet staining

For CV staining, CRC cell lines HCT116 and HT29 were seeded at 1 × 10^6^ cells per well in 6-well culture plates to ensure uniform cellular adherence. After 24 hours, the cells were exposed to increasing concentrations of the antagonist (FH535; 3.75, 7.5, 15, 30, and 60 µM), with untreated wells serving as negative controls. Following a 24-hour treatment period, cells were fixed with methanol and stained using 0.5% (w/v) CV solution. This process allowed for cell fixation and visualization of stained cells. To quantify the stained cells, the retained dye was solubilized, and the absorbance was measured at 570 nm using a spectrophotometer.

### 2.6 Annexin V-FITC apoptosis staining assay

CRC cells (HCT116 and HT29) were seeded at 1 × 10^5^ cells/mL and treated with the Wnt antagonist FH535 (10, 20, and 40 µM) for 24 hours. Following treatment, cells were harvested and stained with Annexin V-FITC and PI using a commercially available apoptosis detection kit (ab14085 abcam) according to the manufacturer’s instructions. A total of 50,000 events per sample were collected for analysis. Fluorescence compensation was performed using single-color controls for Annexin V-FITC and PI. Flow cytometric analysis was conducted using a FACS Aria III flow cytometer (BD Biosciences), and the data were analyzed with FlowJo software.

### 2.7 Cell cycle analysis

CRC cells (1 × 10^5^) were cultured in six-well plates and treated with the Wnt antagonist (FH535; 10, 20, and 40 µM) for 24 hours. After treatment, cells were harvested, centrifuged to obtain pellets, and fixed overnight in 1 mL of 75% ethanol at -20 °C. The following day, cells were washed and treated with RNase A to remove RNA, then stained with PI for 20 minutes at room temperature in the dark. The samples were analyzed using a BD Accuri C6 flow cytometer (Becton, Dickinson and Company, Franklin Lakes, NJ, USA). PI fluorescence was measured using the FL3 detector (PI channel) in linear mode. Doublet discrimination was performed by comparing PI-area vs. height signals. Data were analyzed using FlowJo Software (FlowJo LLC, Ashland, OR, USA).

### 2.8 Western blotting

CRC cells (1x10^6^ cells/mL) were seeded and treated with either Wnt antagonist (FH535) in a dose-dependent manner (10, 20, and 40 µM) for a 24-hour time point. Following treatment, cells were collected and lysed with RIPA lysis buffer (Thermo Fisher Scientific). Whole-cell protein lysates were quantified using the Bradford protein assay (Bio-Rad) with protein Assay Dye Reagent (500-0006, Bio-Rad), and absorbance was measured at 595 nm using a spectrophotometer. Lysate aliquots containing 30 μg protein were separated by 10% SDS-PAGE and transferred onto a nitrocellulose membrane (1620112; Bio-Rad). Membranes were blocked with 5% skimmed milk in TBST for 1 hour at room temperature, then incubated overnight at 4 °C with primary antibodies. The following primary antibodies were used: Wnt Signaling Antibody Kit (#2915T- Cell Signaling Technology), β-catenin Signaling Antibody Kit (2951T- Cell Signaling Technology), Caspase-3 (9665-Cell Signaling Technology), MCP-1/CCL2 (ab214819, Abcam), and MCP-2/CCL8 (ab155967, Abcam). After washing, the membranes were incubated with appropriate secondary antibodies (anti-mouse and anti-rabbit HRP-conjugated; 7076 and 7074, Cell Signaling Technology) for one hour at room temperature. Protein bands were visualized using an ECL detection kit (32106, ThermoScientific Pierce). Band intensities were quantified using Bio-Rad Image Lab software with variable exposure times. Tubulin was used as the loading control for normalization.

### 2.9 Wound healing assay

To assess the migratory potential of antagonist (FH535)-treated CRC cells, a wound healing (scratch) assay was conducted. In brief, HCT116 and HT29 cells (5 × 10^6^) were seeded into 6-well culture plates and allowed to adhere until a confluent monolayer formed. To minimize the contribution of cell proliferation to wound closure, cells were pre-treated with mitomycin C (10 µg/mL) for at least 2 h at 37 °C prior to scratch formation. Subsequently, a uniform scratch was introduced across the cell monolayer using a sterile 10 μL micropipette tip. The wells were gently rinsed with phosphate-buffered saline (PBS) to remove cellular debris generated during wounding. Initial images (time point 0 h) were acquired prior to FH535 treatment. Cells were then exposed to varying concentrations (10, 20, and 40 µM) of FH535 for 24 hours, while an untreated group served as the control. At the end of the treatment period (24 hours), images of the wound area were captured, and the extent of wound closure was quantified using ImageJ software. Cell migration was quantified as a percentage of wound closure relative to the untreated control.

### 2.10 Boyden chamber invasion assay

The impact of the antagonist FH535 on CRC cell invasiveness was evaluated using a previously established protocol with minor modifications. In brief, 0.5 × 10^6^ HCT116 and HT29 cells were seeded into the upper chamber inserts of a 6-well Boyden chamber system pre-coated with Matrigel to simulate the extracellular matrix barrier. Cells were exposed to varying concentrations (10, 20, and 40 µM) of the FH535 or maintained as untreated controls for 24 hours in serum-free or low-serum medium. The lower chambers were filled with complete medium supplemented with 10% FBS to serve as a chemoattractant, promoting directional cell invasion. During incubation, cells migrated through the Matrigel-coated semipermeable membrane toward the chemoattractant. After 24 hours, the membranes were fixed in methanol and stained with 0.5% CV to visualize the invaded cells. Non-invading cells on the upper surface of the membrane were carefully removed using sterile cotton swabs. The membranes were then inverted into grease-free glass slides, and the stained invasive cells adhering to the underside were examined under a microscope. Quantification was performed by counting stained cells in multiple randomly selected fields, and results were expressed as the percentage of invaded cells relative to the untreated control.

### 2.11 siRNA transfection

RNAiMax (13778150) obtained from ThermoFisher Scientific and SignalSilence^R^ β-catenin siRNA (targeting Homo sapiens β-catenin; 6225S) were procured from Cell Signaling Technology. Briefly, CRC (HCT116 and HT29) cells were seeded in 6-well culture plates (for CV staining and protein analysis via western blotting). Transient transfections were carried out with the SignalSilence^R^ β-catenin siRNA using RNAiMax transfection reagent in accordance with the manufacturer’s instructions for a 48 h timepoint.

### 2.12 Quantitative analysis of cytokine secretion profile

Concentrations of MCP1, MCP2, Tumor Necrosis Factor alpha (TNF-α), Interleukin 10 (IL-10), Interleukin 6 (IL-6) and Transforming Growth Factor beta (TGF-β) released in cell culture supernatants were measured using ELISA kits (MCP1 #ab179886, MCP2 #ab100602, TNF-α #ab46087, IL-10 #ab46034, #ab46027, TGF-β #ab100647; Abcam (Cambridge, UK) following the manufacturer’s instructions.

### 2.13 Real-time quantitative polymerase chain reaction analysis

For real-time quantitative reverse transcription polymerase chain reaction (RT-qPCR), total RNA was extracted using a PureLink RNA Mini Kit (Thermo Fisher Scientific, Waltham, MA, USA). cDNA synthesis was performed using a High-Capacity RNA-to-cDNA Kit (Thermo Fisher Scientific, Waltham, MA, USA) with a GeneAmp PCR System 9700 thermal cycler (Thermo Fisher Scientific, Waltham, MA, USA). RT-qPCR analyses were performed using the iTaq Universal SYBR Green Supermix (Bio-Rad, Hercules, CA, USA) with the CFX Connect (Bio-Rad, Hercules, CA, USA). Primers for MCP1 Forward: 5′CAGCCAGATGCAATCAATGCC-3′**;** Reverse: 5′-TGGAATCCTGAACCCACTTCT-3′ and MCP2 Forward: 5′-CCTGCTGCTTTGCCTACCTT-3′; Reverse: 5′-GGTGTCTGGGTTGAGGGTCT-3′. All experiments were performed in accordance with the manufacturer’s instructions. The expression of target genes was normalized to the GAPDH reference gene and analyzed using the 2−^ΔΔCt^ method.

### 2.14 In-silico analysis

In-silico analysis was conducted using the UALCAN (https://ualcan.path.uab.edu/cgi-bin/ualcan-res.pl) and the TIMER2.0 website (https://cistrome.shinyapps.io/timer/) to assess the expression of CTNNB1, MCP1, and MCP2, perform gene expression correlational analysis, patient survival analysis, and examine gene-related leukocyte infiltration levels.

### 2.15 Statistical analysis

All experiments included at least three biological replicates (n = 3). Data are shown as mean ± standard deviation (SD) or standard error of the mean (SEM), as specified in the figure legends. Comparisons between two groups used an unpaired two-tailed Student’s t-test. Analyses of multiple groups used one-way ANOVA with Tukey’s *post hoc* test. Effect sizes and 95% confidence intervals are reported where applicable. Statistical significance was defined as p < 0.05.

## 3 Results

### 3.1 FH535 reduces CRC cell viability

**Figure 1:**
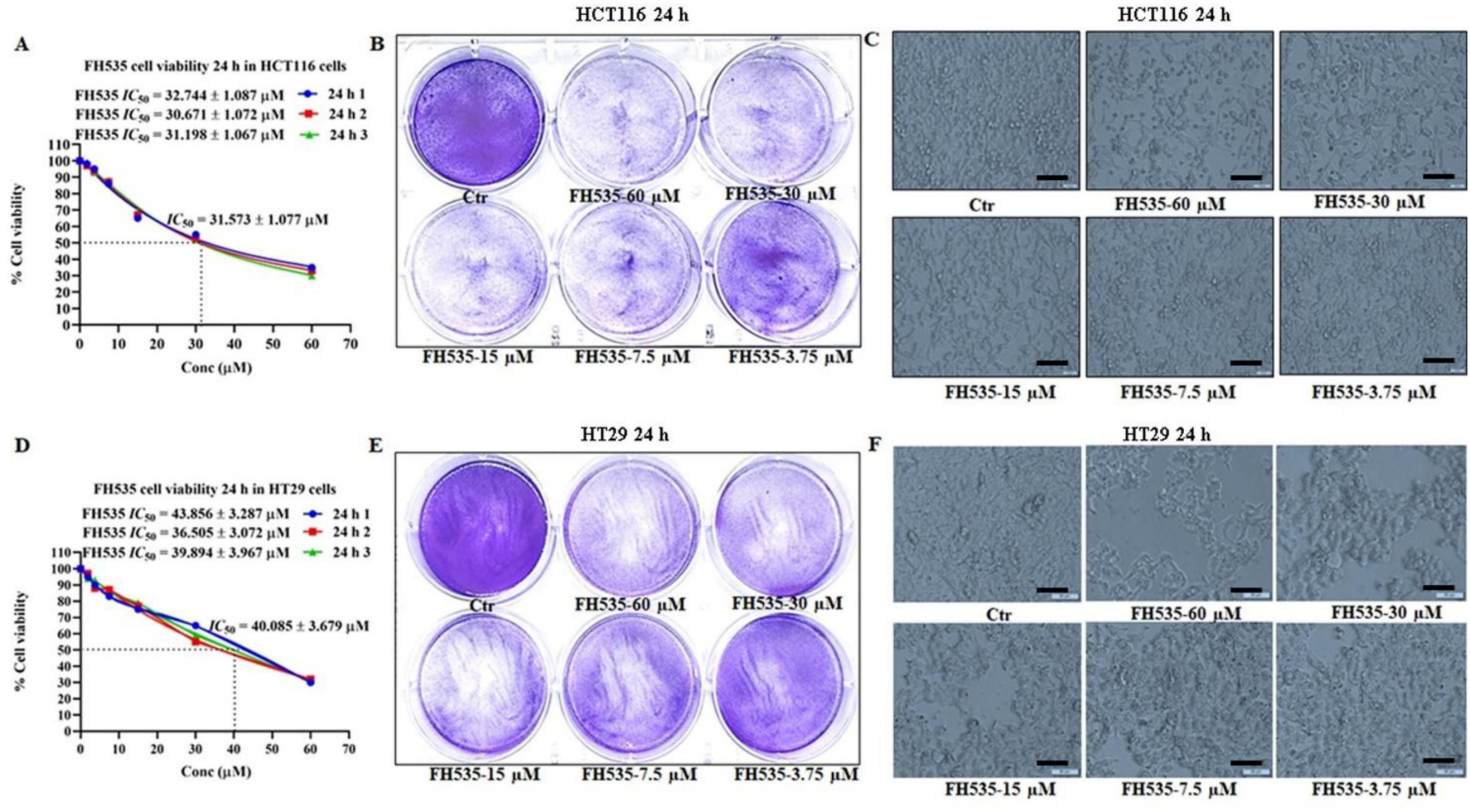
Effect of the Wnt antagonist FH535 on CRC cell viability as assessed by MTT, CV staining, and phase-contrast microscopy. **(A, D)** MTT assay of HCT116 and HT29 cells treated with increasing concentrations of FH535 (10, 20, 30, 40, 50, 60, and 70 μM) for 24 hours. **(B, E)** CV staining of HCT116 and HT29 cells following FH535 treatment at concentrations of 3.75, 7.5, 15, 30, and 60 μM for 24 hours. **(C, F)** Phase-contrast microscopy images showing morphological alterations in HCT116 and HT29 cells treated with the same concentrations of FH535 for 24 hours. Magnification 40x, scale bar 50 µm. Data represent the mean ± SEM of three (n=3) independent experiments.

To determine the IC_50_ concentration of the Wnt antagonist (FH535) for subsequent use, an MTT assay was performed (**Figure 1**) on CRC (HCT116 and HT29) cells following treatment with FH535, a β-catenin/Tcf inhibitor, at 10, 20, 30, 40, 50, 60, and 70 μM for 24 hours. Results demonstrated that CRC cell viability (**Figures 1A, D**) decreased in a dose-dependent manner with an IC_50_ value of 31.573 ± 1.077 µM and 40.085 ± 3.679 µM for HCT116 and HT29 cells, respectively. Furthermore, CV staining of HCT116 and HT29 cells in 6-well plates following treatment with increasing concentrations of FH535 demonstrated a marked dose-dependent reduction in cell viability (**Figures 1B, E**). Additionally, phase-contrast microscopy revealed a markedly increased number of rounded, non-adherent (dead or dying) cells in both HCT116 and HT29 cultures treated with high concentrations of FH535 (30 and 60 µM) compared to untreated control (**Figures 1C, F**).

### 3.2 FH535 treatment disrupts CRC cell cycling and induces apoptosis in CRC cells

**Figure 2:**
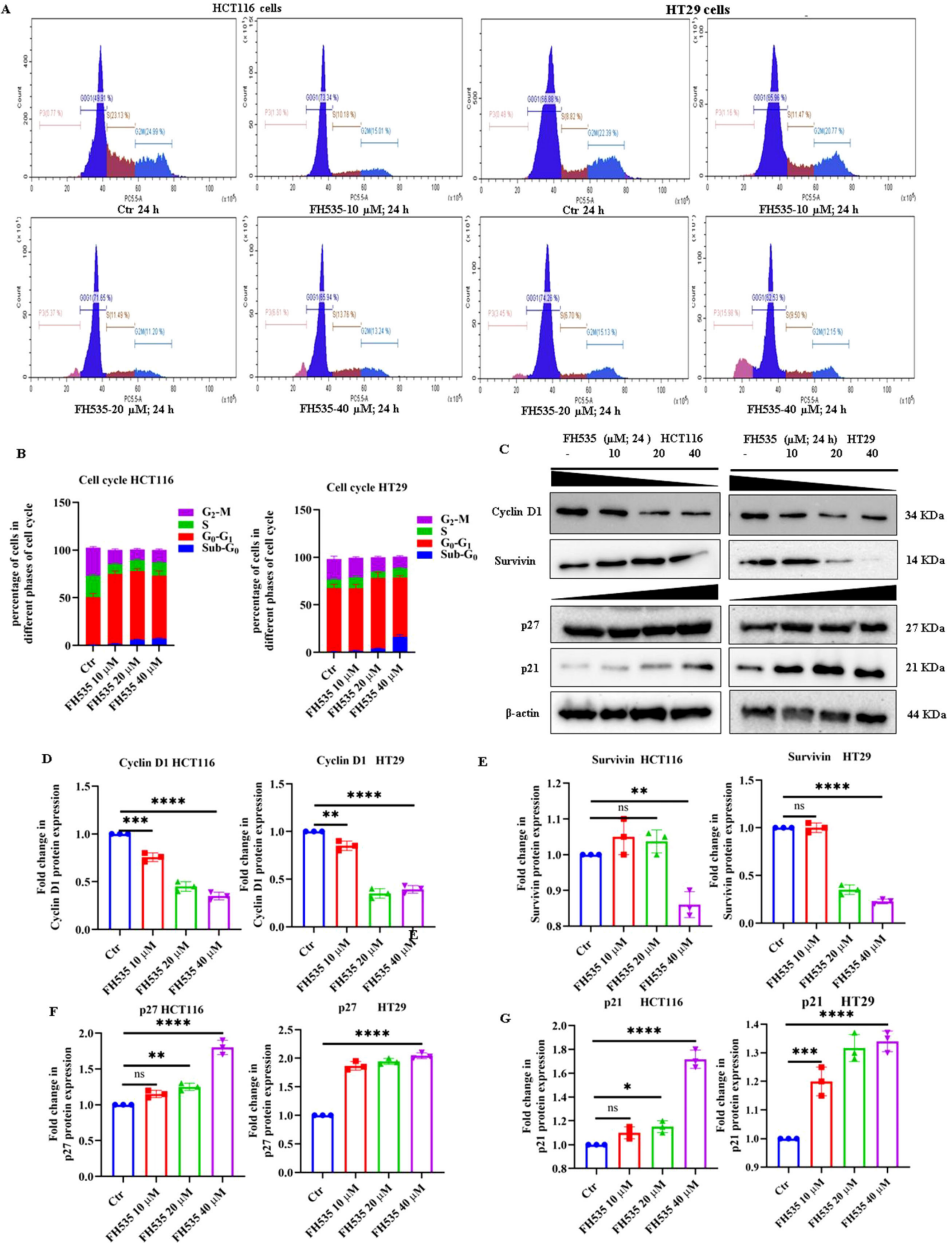
Analysis of cell cycle dynamics and expression of cell cycle regulatory proteins in FH535-treated HCT116 and HT29 cells. **(A)** Representative flow cytometry histograms depicting cell cycle distribution in HCT116 and HT29 cells following treatment with FH535 (10, 20, and 40 µM) for 24 hours compared to untreated controls. **(B)** Quantitative bar graph showing propidium iodide (PI) mean fluorescence intensity for cell cycle phases of treated versus control HCT116 and HT29 cells. **(C)** Immunoblot analysis of checkpoint regulators (p21 and p27) and key cell cycle proteins (cyclin D1 and survivin) in HCT116 and HT29 cell lysates treated with FH535 at the indicated concentrations for 24 hours. Densitometric quantification of protein expression levels showing fold changes in **(D)** cyclin D1, **(E)** survivin, **(F)** p27, and **(G)** p21 relative to untreated controls. Data are presented as mean ± SEM of at least three (n=3) independent experiments: *p < 0.05, **p < 0.01, ***p < 0.001, ****p < 0.0001.

We next investigated the impact of FH535 treatment on cell cycle progression in CRC (HCT116 and HT29) cells by exposing them to increasing concentrations of FH535 (10, 20, and 40 µM) for 24 hours, followed by flow cytometric analysis of cell cycle. As depicted in (**Figures 2A, B**), FH535-treated HCT116 and HT29 cells exhibited a marked accumulation in the pro-apoptotic/apoptotic SubG1 phase compared to untreated controls (**Figure 2B**). This shift was associated with a substantial downregulation in expression of key cell cycle regulatory proteins, including cyclin D1 and survivin, and a concomitant upregulation of checkpoint inhibitors p21 and p27, particularly at high FH535 concentrations (20 and 40 µM) (**Figures 2C–G**).

**Figure 3:**
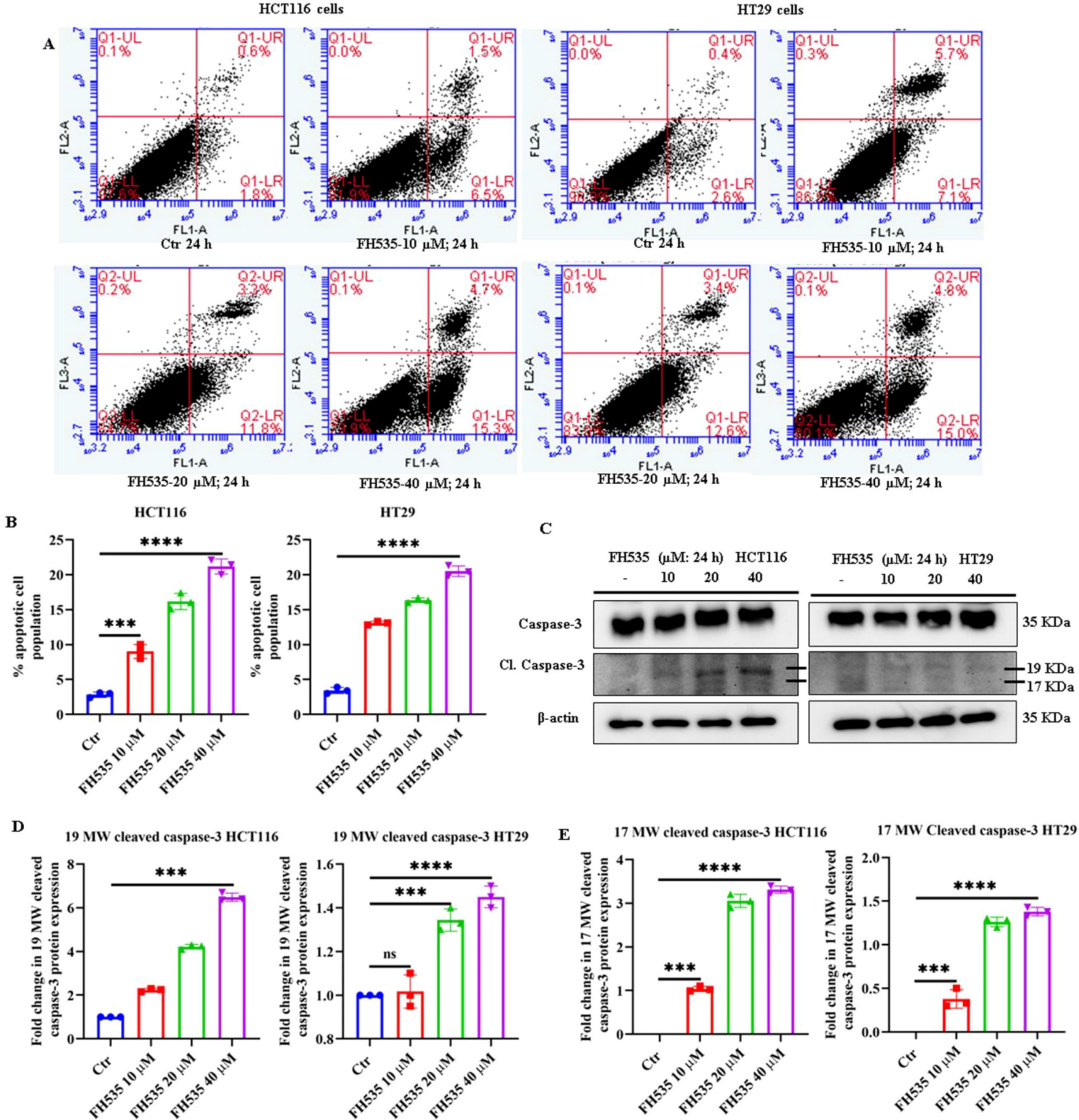
FH535 induces apoptotic cell death in HCT116 and HT29 cells. **(A)** Representative flow cytometry plots of Annexin V-FITC and propidium iodide (PI) dual staining in HCT116 and HT29 cells following treatment with FH535 at 10, 20, and 40 µM for 24 hours. **(B)** Quantitative analysis of apoptotic cell populations (early and late apoptosis; quadrants Q2 and Q4) derived from Annexin V-FITC/PI staining of treated vs control HCT116 and HT29 cells. **(C)** Immunoblot analysis showing the expression of caspase-3 and its cleaved fragments (MW 17 and 19) in HCT116 and HT29 cells treated with FH535 (10, 20, and 40 µM) for 24 hours. Densitometric quantification of protein expression levels showing fold changes in **(D)** cleaved caspase-3 (MW-17), and **(E)** cleaved caspase-3 (MW-19) fragments relative to untreated control of HCT116 and HT29 cells. Data are expressed as mean ± SE of at least three (n=3) independent experiments: ***p < 0.001, ****p < 0.0001, ns, not significant.

To further elucidate the mechanism of FH535-induced cell death, we performed Annexin V-FITC staining to quantify apoptotic populations of HCT116 and HT29 cells. A dose-dependent increase in apoptosis was observed, with 8.0%, 15.1%, and 20.0% apoptotic cells detected following treatment of HCT116 cells with 10, 20, and 40 µM FH535, respectively, compared with 2.4% in untreated cells (**Figures 3A, B**). Whereas 12.8%, 16.0%, and 19.8% of apoptotic cells were detected following treatment of HT29 cells with 10, 20, and 40 µM of FH535, respectively, as compared to 3.0% in untreated cells (**Figures 3A, B**). Immunoblotting analyses consistently revealed prominent caspase-3 cleavage (MW 17 and 19) in lysates from cells treated with high FH535 doses, indicating activation of apoptotic signaling pathways at higher doses in both HCT116 and HT29 cells (**Figures 3C–E**). In short, FH535 appears to disrupt cell cycle progression and induce apoptosis in CRC cells, suggesting its potential therapeutic utility in targeting Wnt/β-catenin-driven colorectal tumorigenesis.

### 3.3 FH535 treatment reduces CRC cell migration and invasive potential

**Figure 4:**
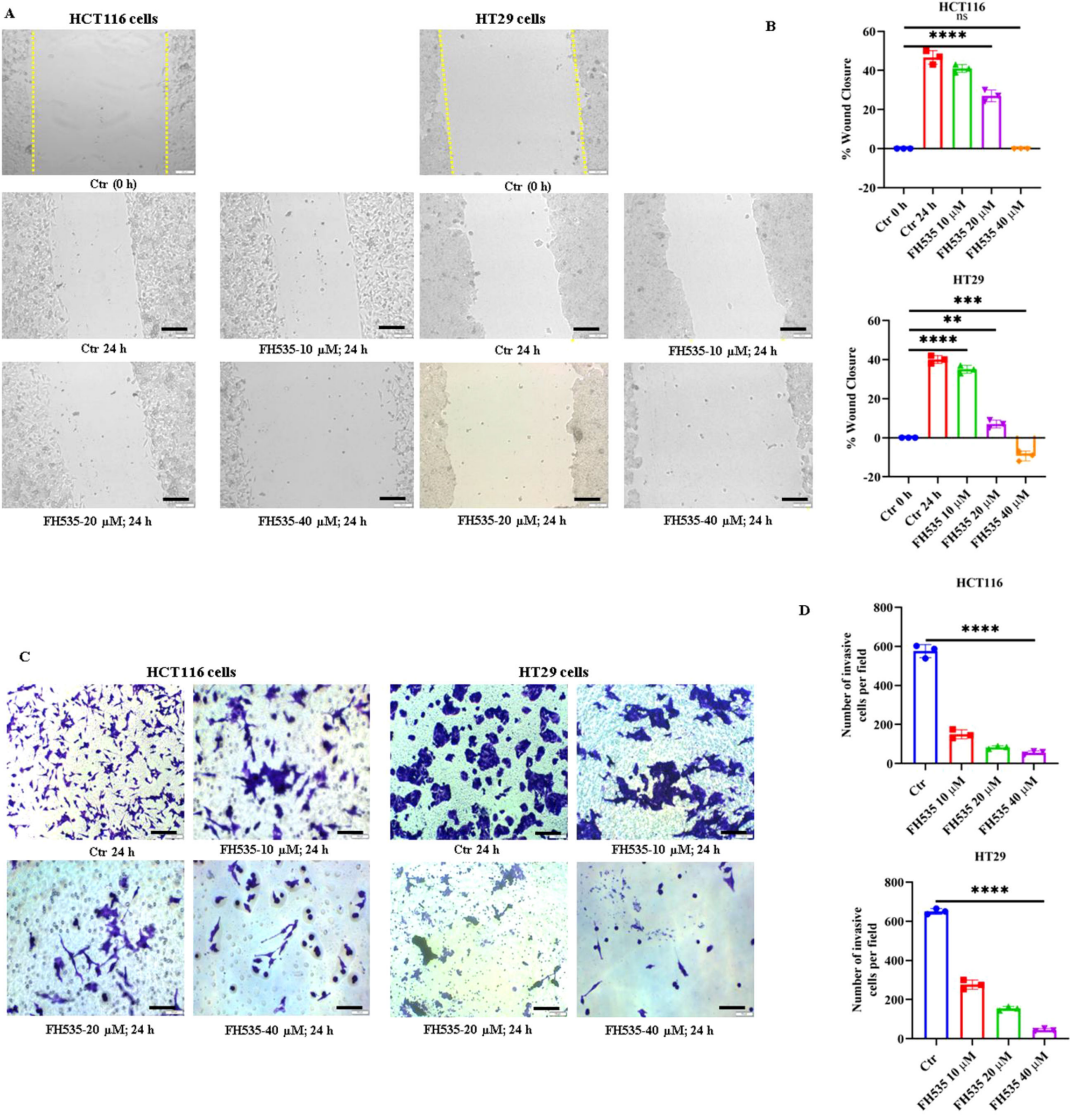
FH535 attenuates the migratory and invasive capacity of CRC cells. **(A)** Wound healing assay images demonstrating impaired migration of HCT116 and HT29 cells treated with FH535 for 24 hours in a dose-dependent manner (magnification 10x, scale bar 100 µm). **(B)** Bar graph depicting the percentage inhibition of cell migration relative to controls after FH535 exposure of HCT116 and HT29 cells for 24 hours. **(C)** Crystal violet-stained micrographs (40× magnification, scale bar 100 µm) illustrating the reduced invasion of CRC (HCT116 and HT29) cells treated with FH535 (10, 20, and 40 µM) for 24 hours compared to controls. **(D)** Quantification of invasion inhibition, expressed as percentage reduction in invasive cells (counted fields n>10). Data are presented as mean ± SEM of at least three (n=3) independent experiments; **p ≤ 0.01, ***p < 0.001, ****p < 0.0001.

To further assess the anti-growth effects of FH535, the wound healing potential of FH535-treated HCT116 and HT29 cells was examined. As shown in **Figures 4A, B**, FH535 treatment significantly reduced the wound-healing potential (migratory capacity) of both HCT116 and HT29 cells at 20 and 40 µM FH535 concentrations.

The invasive potential of HCT116 and HT29 cells under FH535 treatment was further evaluated using a Boyden chamber assay. Cells were treated with FH535 (10, 20, and 40 µM) for 24 hours, and their ability to migrate through a Matrigel-coated semipermeable membrane towards a chemoattractant (10% FBS in the lower chamber) was assessed. As shown in **Figures 4C, D**, FH535 treatment led to a significant, dose-dependent reduction in the number of invasive cells compared with the untreated control, indicating promising anti-invasive activity.

### 3.4 FH535-induced inhibition of Wnt/β-catenin exerts immunomodulatory effects on the CRC TME

**Figure 5:**
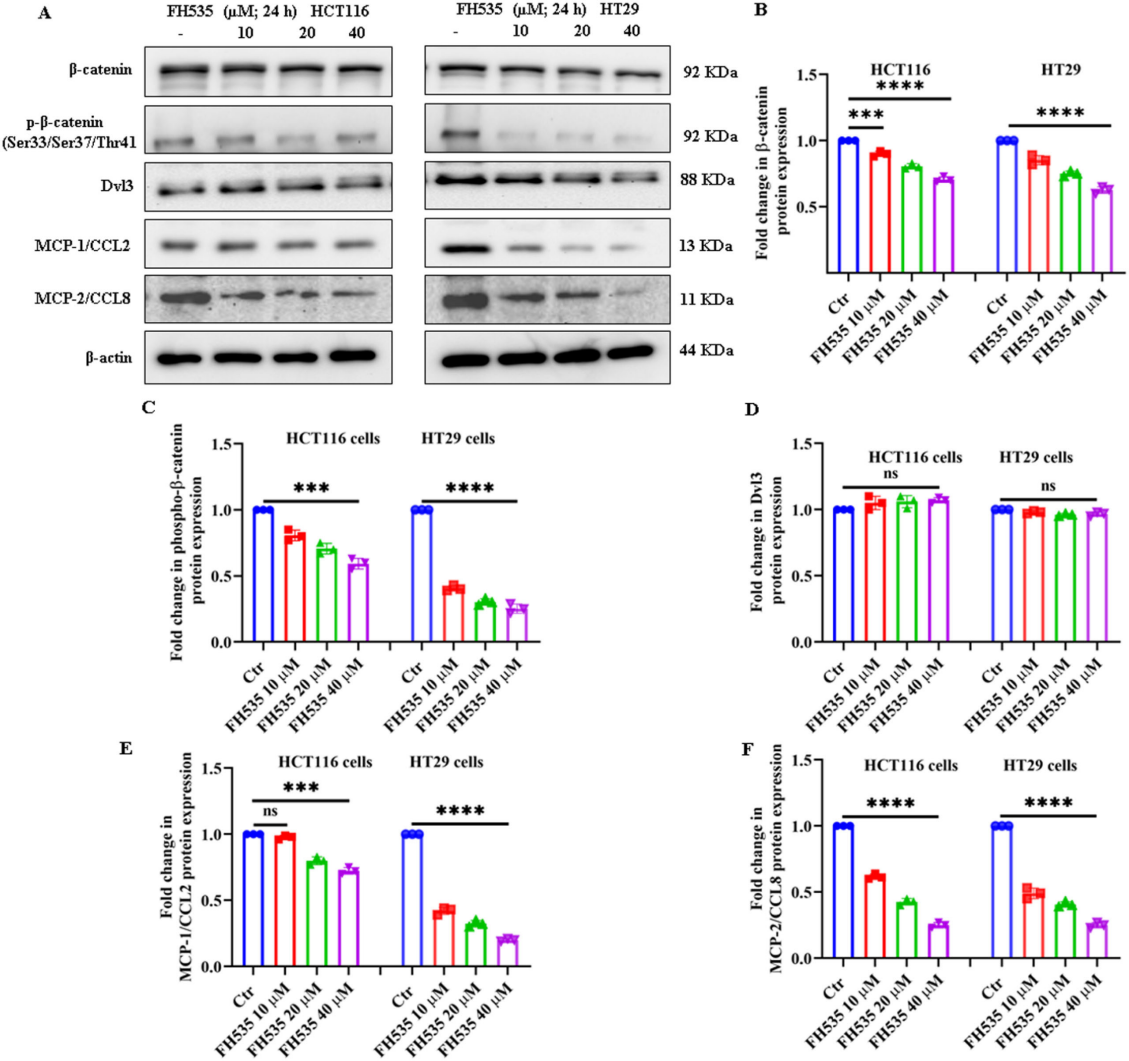
The inhibitory effects of FH535 on the Wnt/β-catenin signaling pathway in CRC cells. **(A)** Representative immune-blot expression of key Wnt/β-catenin and downstream signaling proteins of HCT116 and HT29 cells treated with varying doses of FH535 (10, 20, and 40 µM) and untreated control. Densitometric quantification of protein expression levels showing fold changes in **(B)** total β-catenin, **(C)** p-β-catenin (Ser33/Ser37/Thr41), **(D)** Dvl3, **(E)** MCP1/CCL2, and **(F)** MCP2/CCL8 protein expression relative to untreated controls. Data are expressed as mean ± SE of a minimum of three (n=3) independent experiments, ***p < 0.001, ****p < 0.0001, ns, not significant.

HCT116 and HT29 CRC cells treated with varying concentrations of FH535 were assessed for the expression of some key components of the Wnt/β-catenin pathway. As shown in (**Figures 5A–C**), a significant reduction in both total and phosphorylated β-catenin (p-β-catenin-Ser33/37/Thr41) was observed in HCT116 and HT29 cells relative to untreated controls at 24 hours post-treatment with FH535. No significant change was observed in the expression of Dvl3 protein (**Figures 5A–D**) relative to untreated controls. Interestingly, FH535-treated HCT116 and HT29 cells showed a significant, dose-dependent decrease in the expression of both MCP1/CCL2 and MCP2/CCL8. This reduction occurred at both the translational (**Figures 5A, E, F**) and transcriptional (**Supplementary Figures 1A, B**) levels in HCT116 and HT29 cells. Furthermore, ELISA assay results showed a marked reduction in MCP1 and MCP2 in the conditioned media of HCT116 and HT29 cells treated with varying doses of FH535 (**Supplementary Figures 1C, D**). These results demonstrate that FH535 suppresses Wnt/β-catenin signaling in CRC cells by reducing total and phosphorylated β-catenin (Ser33/37/Thr41) levels and downregulating pro-inflammatory chemokine production (MCP1/CCL2 and MCP2/CCL8) in secretion as well as at the transcriptional and translational levels. To confirm whether the conditional media of FH535-treated HCT116 and HT29 cells could polarize THP-1 cells to M1 or M2 macrophages to modulate a TME-like environment, we treated monocyte-like THP-1 cells with the conditional media obtained from FH535-treated HCT116 and HT29 cells. After 24 h of exposure, FH535-treated conditional media can partially polarize THP-1 cells to become more adherent with large dendritic-like macrophages (**Supplementary Figures 2A, B**). To check whether THP-1 cells polarize to M1 or M2 macrophages, we perform ELISA assays for key cytokine markers of M1 and M2 macrophages. As shown in (**Supplementary Figures 2C, D**), we observe an increased expression of TNF-α and IL-6-like cytokine markers, which are mostly secreted by M1 macrophages, and a subsequent decrease in expression of TGF-β and IL-10-like cytokine markers of M2 macrophages, suggesting that FH535-treated conditional media has the ability to partially polarize THP-1 monocytes to M1 like macrophages, thereby modulating a TME.

**Figure 6:**
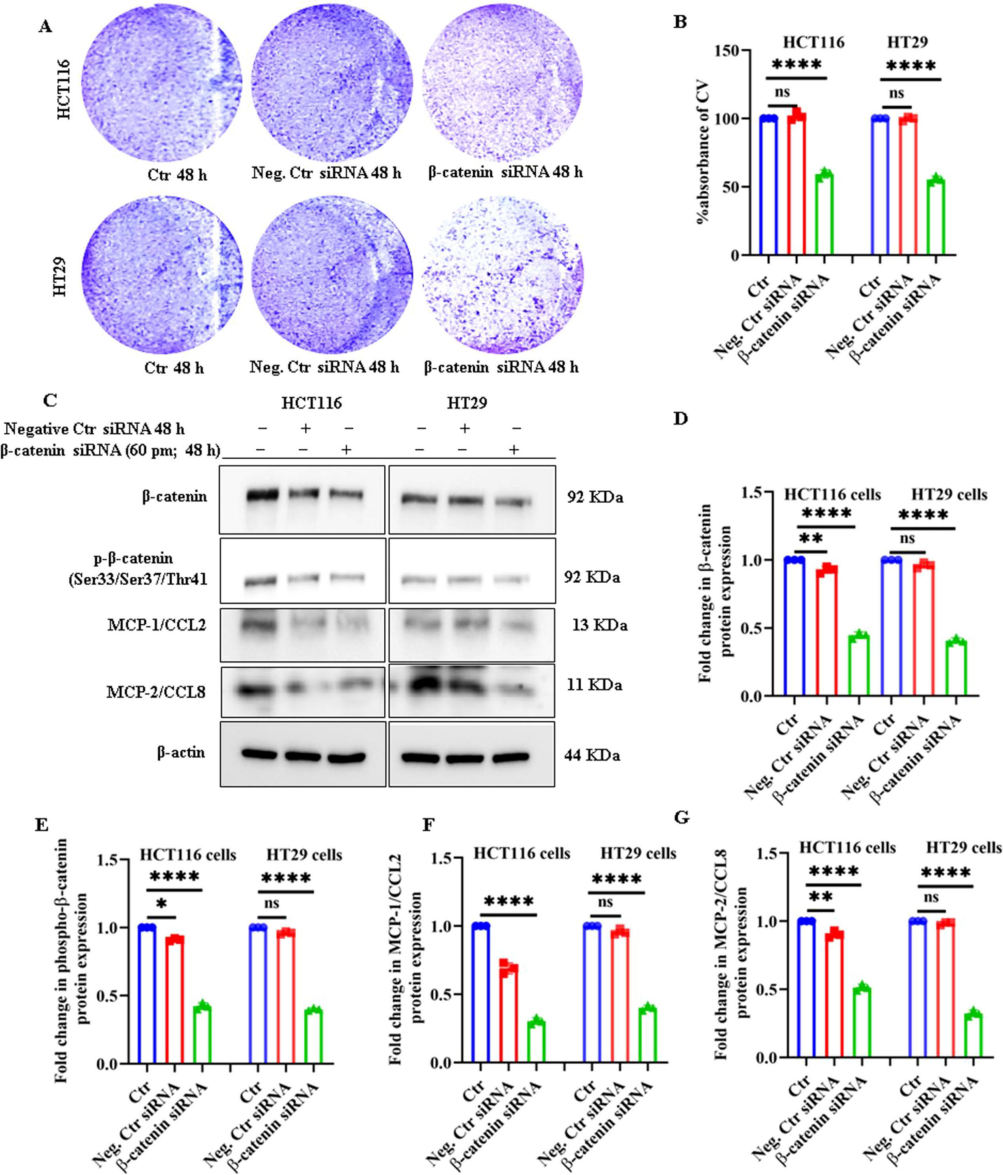
Silencing of β-catenin suppresses Wnt/β-catenin signaling in CRC cells. **(A)** Representative CV staining images illustrating reduced cell viability in β-catenin siRNA-transfected HCT116 and HT29 cells compared to control siRNA-transfected and untreated controls. **(B)** Quantitative analysis of CV stain absorbance showing the percentage of viable cells following β-catenin and control siRNA transfection in HCT116 and HT29 cells over 48 h. **(C)** Representative immunoblot images depicting the expression levels of key Wnt/β-catenin pathway and downstream effector proteins in HCT116 and HT29 cells post-transfection with β-catenin siRNA and control siRNA for 48 h. Densitometric analyses showing relative fold changes in the expression of **(D)** total β-catenin, **(E)** phosphorylated β-catenin (p-β-catenin-Ser33/Ser37/Thr41), **(F)** MCP1/CCL2, and **(G)** MCP2/CCL8 proteins in β-catenin knockdown cells compared to control groups. Data represent the mean ± SE from at least three (n=3) independent experiments: *p < 0.05, **p < 0.01, ****p < 0.0001, ns, not significant.

To validate the observation that inhibition of Wnt/β-catenin signaling by FH535 treatment precipitates anti-growth and immunomodulatory effects in CRC cells, the effect of siRNA-based β-catenin-silencing on CRC cell growth was assessed at 48 hours post-β-catenin knockdown. Immunoblotting data confirmed silencing of β-catenin in both cell lines (**Figures 6C, D**). β-catenin knockdown significantly decreased the viability of HCT116 and HT29 cells, as measured by CV staining (**Figures 6A, B**). It also resulted in a significant reduction in p-β-catenin (Ser33/37/Thr41) expression in HCT116 and HT29 cells (**Figures 6C, E**). Moreover, a marked reduction in the expression of MCP1/CCL2 and MCP2/CCL8 proteins was observed in β-catenin-silenced HCT116 and HT29 cells (**Figures 6C, F, G**). These findings further confirm that silencing β-catenin, as with FH535, reduces cell growth and inhibits the synthesis of pro-inflammatory chemokines within the CRC TME.

### 3.5 The regulatory role of CTNNB1 in the expression of MCP1 and MCP2 is context-dependent

**Figure 7:**
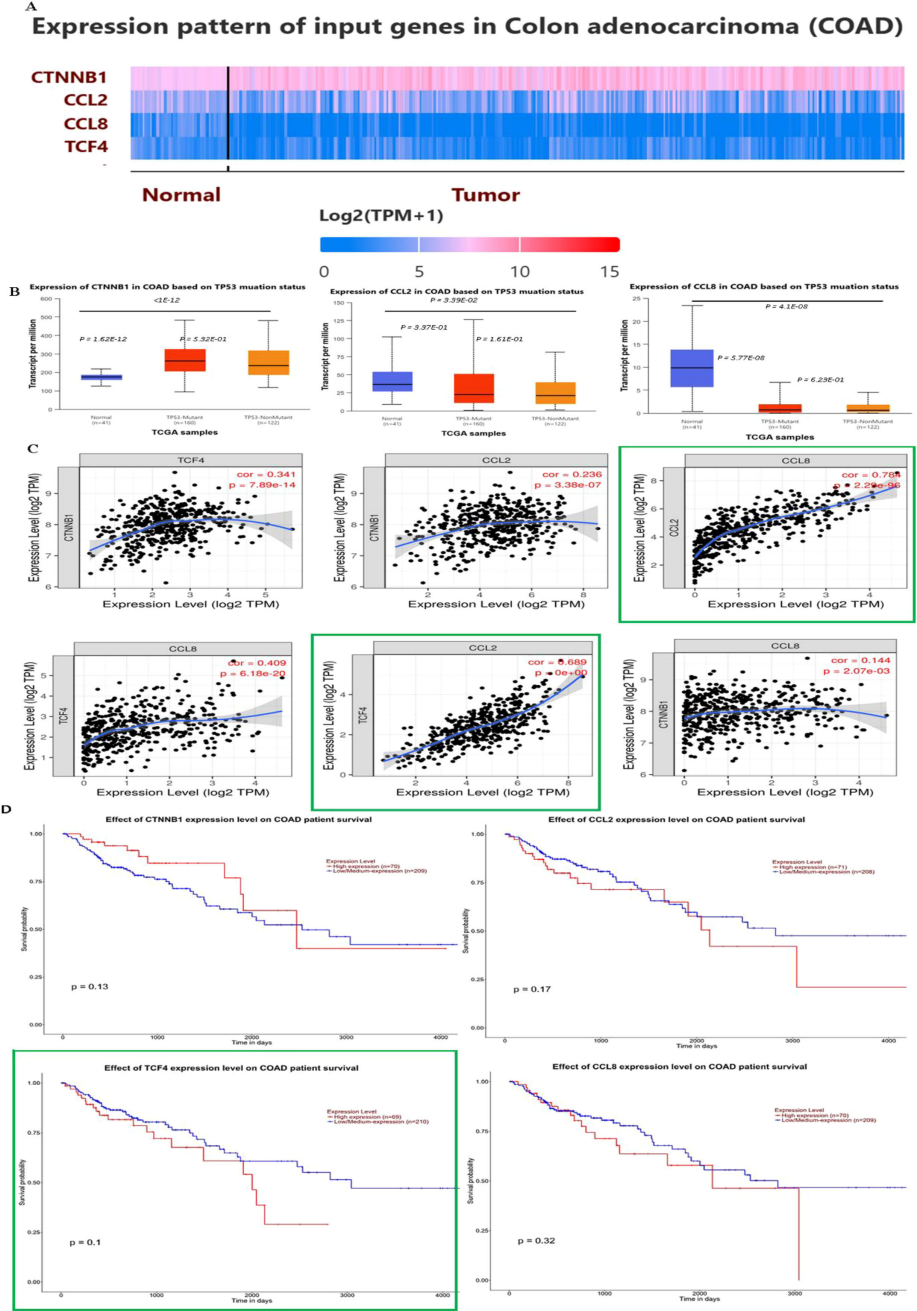
CTNNB1, MCP1, and MCP2 expression, co-expression, and survival-related *insilico* analysis in TP53 wild-type and TP53 mutant COAD tissues. **(A, B)** Expression levels of CTNNB1, TCF4, MCP1, and MCP2 in COAD patients. **(C)** Co-expression analysis of CTNNB1 vs. TCF4, MCP1, or MCP2; TCF4 vs MCP1, or MCP2; and MCP1 vs MCP2.in COAD tissues. **(D)** COAD patient survival analysis in relation to CTNNB1, TCF4, MCP1, and MCP2 expression.

The observation that inhibiting β-catenin downregulates MCP1 and MCP2 expression prompted us to perform a detailed in silico analysis to better understand the immunomodulatory effects of the Wnt/β-catenin pathway. As shown in **Figures 7A, B**, while the expression of CTNNB1 (the gene that encodes β-catenin) is highly upregulated in COAD tissues, that of MCP1 and MCP2 is highly downregulated; no significant differences were observed regarding whether the COAD tissue is TP53 wild type or mutated. Co-expression analysis showed that while CTNBB1 expression does not directly correlate with MCP1 or MCP2 expression (**Figure 7C**), a strong positive correlation was observed between TCF4 expression, which is known to be positively influenced by CTNNB1, and MCP1, but not MCP2. Moreover, a very strong positive correlation was observed between MCP1 and MCP8, possibly suggesting that TCF4-mediated upregulation of MCP1 may enhance MCP8 expression. Upregulation of TCF4 correlated with a significant decrease in overall survival in COAD patients (**Figure 7D**); the associations between upregulation of CTNNB1, MCP1, and MCP2 and decreased overall survival were not statistically significant.

**Figure 8:**
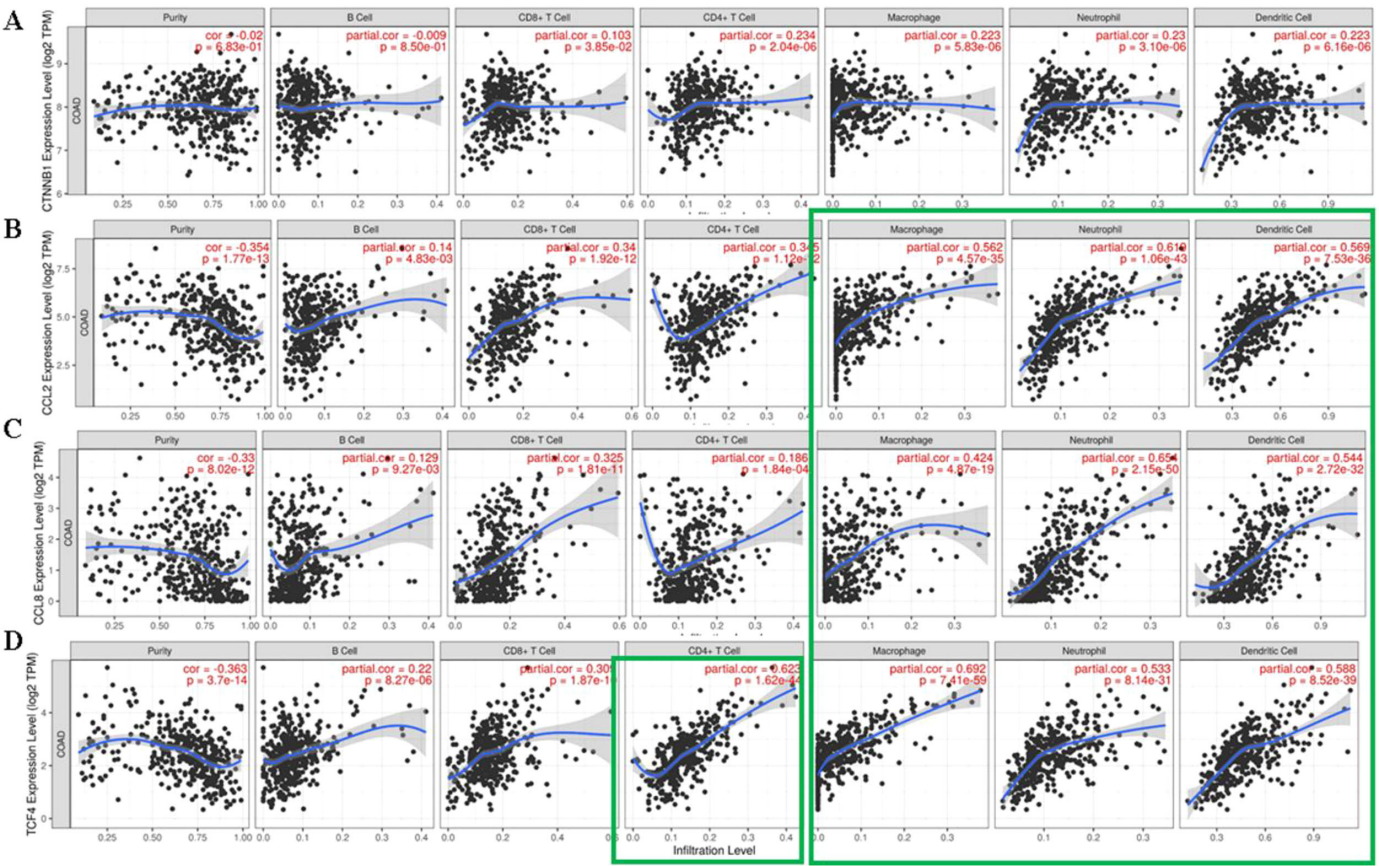
**C**orrelational analysis involving **(A)** CTNNB1, **(B)** MCP1, **(C)** MCP2, and **(D)** TCF4 expression and leukocyte infiltration levels in COAD tissues.

The clinical consequences of increased expression of CTNNB1, TCF4, MCP1, and MCP2 were further analyzed by assessing their association with leukocyte infiltration (**Figure 8**). Increased expression of CTNNB1 did not significantly alter the pattern of leukocyte infiltration within the CRC TME. However, increased expression of MCP1, MCP2, or TCF4 is associated with increased infiltration by macrophages, neutrophils, and dendritic cells (**Figures 8B–D**). In the case of TCF4, an increase in CD4+ T cell infiltration was also observed (**Figure 8D**). Interpreting this data is difficult at this level, as the functional profile of infiltrating cells, pro- or anti-tumor, requires further *in vivo* validation (work in progress).

## 4 Discussion

The Wnt/β-catenin signaling pathway is a highly conserved regulatory cascade that orchestrates embryonic development and tissue homeostasis, while its aberrant activation is strongly implicated in tumorigenesis, including CRC (20). In recent years, components of the Wnt pathway have emerged as reliable biomarkers and potential targets for cancer therapy. In line with this, several small-molecule inhibitors targeting components of the Wnt/β-catenin pathway, such as PR1-724, LGK974, OMP-54F28, and OMP-18R5, have demonstrated therapeutic promise and are undergoing clinical evaluation (21, 22). FH535 represents a unique class of Wnt/β-catenin antagonists that block the recruitment of β-catenin and glutamate receptor-interacting protein 1 (GRIP1), thereby suppressing transcriptional activation of β-catenin/TCF target genes critical for tumor survival and progression (15). Recent reports suggest that FH535 selectively inhibits cancer cells with minimal toxicity to normal tissue (23). FH535’s anti-proliferative effects in CRC and other malignancies are established. This study identifies, for the first time, MCP1/CCL2 and MCP2/CCL8 suppression as downstream effects of β-catenin pathway inhibition. These results expand the understanding of Wnt/β-catenin signaling, highlighting its role in chemokine-mediated tumor–immune crosstalk in addition to its impact on tumor cell proliferation and survival. In precision oncology, connecting oncogenic signaling pathways with downstream inflammatory responses may enable more targeted therapies. β-Catenin modulates chemokines such as MCP1/CCL2 and MCP2/CCL8, potentially influencing immune cell recruitment, microenvironment composition, and treatment outcomes. These chemokines may also serve as complementary biomarkers of pathway activity or therapeutic response in CRC. Future studies should assess β-catenin activity together with chemokine expression in patient-derived samples. This approach may help identify CRC subsets with unique inflammatory and immune profiles and support the development of pathway-informed, biomarker-driven treatment strategies.

Although previous studies have demonstrated that FH535 exhibits potent antiproliferative effects against CRC cells (24). It remains unclear whether inhibition of the Wnt/β-catenin signaling pathway can suppress the production of pro-inflammatory chemokines, such as MCPs, within the TME, which are implicated in facilitating the dissemination of metastatic cells to distant organs, such as the liver. In the present study, we demonstrated that pharmacological inhibition of the Wnt/β-catenin signaling pathway using the small-molecule antagonist FH535 significantly reduced cell viability in both HCT116 and HT29 colorectal cancer cell lines and abrogated their migration and invasive capability. To ensure that reduced wound closure was attributable to impaired migration rather than altered proliferation, cells were pre-treated with mitomycin C prior to scratch induction. This approach minimizes cell division during the assay period, thereby strengthening the conclusion that FH535 treatment directly suppresses migratory behavior. Therefore, the inhibitory effect observed in the wound healing assay is unlikely to be secondary to reduced viability or proliferation. These findings are consistent with previous reports demonstrating the antiproliferative activity of FH535 in CRC and HCC cells by suppressing cell motility through decreases in Snail, matrix metalloproteinases (MMPs), and Vimentin expression (18, 25). Additionally, similar antiproliferative activity was observed in osteosarcoma cells treated with FH535, which downregulated the Wnt/β-catenin pathway via tankyrases (23). Cell cycle dysregulation is a hallmark of cancer progression. Among cell cycle regulators, cyclin D1 interacts with various cyclin-dependent kinases (CDKs) to form a functional complex that activates the transcription factor E2F1, thereby driving the G1-S phase transition (26). Consequently, the downregulation of Cyclin D1 expression precedes G1-S phase arrest (27, 28). Survivin, on the other hand, regulates critical signaling pathways that promote tumor cell proliferation and confer resistance to apoptosis (29). Our findings indicate that FH535 induces cell cycle arrest in CRC cells, resulting in increased sub-G1 and decreased G2-M and S phase populations. Immunoblot analysis consistently demonstrated a dose-dependent downregulation of key cell cycle regulatory proteins, including cyclin D1 and survivin, and an upregulation of cell cycle checkpoint inhibitors p21 and p27 in CRC cells. This study shows that FH535 consistently blocks cell growth, disrupts the cell cycle, and triggers cell death at MTT-based IC_50_ concentrations. We did not measure all mechanistic endpoints over full time courses or create detailed dose–response curves. Instead, we chose specific treatment times and concentration ranges to assess pathway involvement and downstream effects. This approach provided insight into mechanisms but did not yield kinetic data or endpoint-specific EC_50_/IC_50_ values for precise potency and timing. Future studies should include systematic analyses over time and across endpoints to improve translational and pharmacological understanding of FH535.Furthermore, FH535 triggered apoptosis via activation of the intrinsic or extrinsic pathways, converging on the activation and cleavage of caspase-3, a central effector protease in the apoptotic cascade. Notably, caspase-3 is infrequently mutated in CRC (30), and serves as a reliable marker of apoptotic activation; these findings substantiate the proapoptotic efficacy of FH535. Prior studies have validated the specificity of antibodies recognizing cleaved caspase-3, with no cross-reactivity to its full-length precursor, as confirmed by immunoblotting (31–33). Moreover, numerous small-molecule inhibitors have been shown to induce apoptosis in CRC cells by activating caspase-3, which executes apoptosis by proteolytically degrading a wide range of substrates (34–37). In line with these findings, our data confirmed that FH535 treatment induces dose-dependent caspase-3 activation, as evidenced by the presence of cleaved caspase-3 fragments on immunoblots and an increased proportion of annexin V-FITC–positive apoptotic cells detected by flow cytometry.

Growing evidence supports the critical role of aberrant activation of the Wnt/β-catenin signaling pathway in tumor immune evasion and malignant progression. Upregulation and activation of Wnt/β-catenin signaling initiate LEF1 transcriptional activity, which enhances cancer cell invasion (38, 39) and has been documented as a predictor of poor prognosis in CRC patients (40). Previous studies demonstrated that FH535 treatment significantly downregulated β-catenin at both the transcriptional and translational levels in DLD-1, SW620 (24), and SW480 (25) CRC cells. A key limitation is the specificity of FH535. Although FH535 inhibits canonical Wnt/β-catenin signaling, it also targets PPARs, raising questions about pathway specificity and complicating the interpretation of downstream effects, such as chemokine expression. Therefore, changes in chemokine levels following FH535 treatment cannot be attributed solely to Wnt pathway inhibition. While siRNA-mediated β-catenin knockdown provides supporting evidence, further validation is needed to strengthen causal inference. Using selective Wnt inhibitors, CRISPR/Cas9 gene editing, or genetic rescue experiments to restore β-catenin activity would help confirm pathway specificity and reduce the likelihood of off-target or compensatory effects. Consistent with previous studies, FH535 treatment, and siRNA-based silencing of β-catenin in HCT116 and HT29 CRC cells produced a similar pattern of inhibition of total and phosphorylated β-catenin (Ser33/37/Thr41). Changes in phosphorylated β-catenin (p-β-catenin) indicate alterations at the N-terminal Ser33/Ser37/Thr41 residues, as the phospho-specific antibody targets these sites. Since these residues are essential for β-catenin degradation, reduced signal suggests β-catenin stabilization rather than nonspecific effects. However, Dvl3 protein expression remained unchanged and consistent with that of untreated control cells.

Beyond its direct anti-tumor effects, Wnt/β-catenin signaling is increasingly recognized as a regulator of immune evasion within the TME. Emerging evidence indicates that Wnt/β-catenin signaling suppresses macrophage polarization toward the pro-inflammatory M1 phenotype and promotes M2-like polarization by inducing specific Wnt target genes (41). Tumor-derived chemokines such as MCP1/CCL2, MCP2/CCL8, and MCP3/CCL7 facilitate macrophage recruitment into the TME (42). Our findings demonstrate that inhibition of β-catenin signaling by FH535 markedly decreased MCP1/CCL2 and MCP2/CCL8 protein levels in HCT116 and HT29 cells. Silencing CTNNB1 using siRNA reproduced this suppression, confirming that these chemokines are transcriptionally regulated by the β-catenin pathway. These data suggest that FH535 not only acts as a direct antiproliferative agent but also has secondary immunomodulatory effects by limiting macrophage-attracting chemokine production, thereby potentially counteracting tumor-associated immune suppression. Our data demonstrate that β-catenin signaling regulates the expression of MCP1 and MCP2 in CRC cells. While we did not evaluate monocyte or macrophage recruitment or immune modulation in functional assays *in vivo* settings, to support further investigation into their effects on the tumor immune microenvironment and combined immunotherapy approaches. This work provides a basis for future mechanistic and translational studies. However, the present findings demonstrate that FH535 markedly suppresses MCP1 and MCP2 expression at both translational and transcriptional levels in HCT116 and HT29 cells, indicating effective inhibition of pro-inflammatory chemokine signaling. Reduced secretion of these chemokines in conditioned media further confirms the functional impact of FH535 on the tumor secretome. Importantly, exposure of THP-1 monocytes to FH535-treated conditioned media promoted partial polarization toward an M1-like phenotype, as evidenced by increased TNF-α and IL-6 and decreased TGF-β and IL-10 expression. These results suggest that FH535 not only alters tumor cell signaling but also reshapes the tumor microenvironment (TME) by favoring a pro-inflammatory, anti-tumor macrophage profile. Collectively, this highlights the potential of FH535 to modulate immune components within the TME and enhance anti-tumor responses. To further clarify the regulatory interplay between β-catenin signaling and chemokine expression, in-silico analysis was conducted using publicly available COAD datasets. CTNNB1 expressions were significantly elevated in tumor tissues, whereas MCP1 and MCP2 levels were markedly reduced, regardless of TP53 mutational status. Co-expression analysis revealed no direct correlation between CTNNB1 and MCP1 or MCP2; however, a strong positive correlation was observed between TCF4, an established downstream transcriptional factor of β-catenin, and MCP1, but not MCP2. Interestingly, a robust positive association between MCP1 and MCP2 expression was detected, suggesting that upregulation of MCP1 via TCF4 activation may indirectly enhance MCP2 expression via shared regulatory pathways. Notably, high TCF4 expression correlated with reduced overall survival in COAD patients, whereas increased CTNNB1, MCP1, or MCP2 alone did not show a statistically significant association with patient prognosis. While stromal and immune cells, rather than malignant epithelial cells, primarily drive chemokine expression in tumors. The observed downregulation of MCP1 and MCP2 in bulk tumor datasets does not conflict with previous reports of elevated chemokine levels in the tumor microenvironment. These findings underscore the importance of using spatially resolved and subtype-stratified analyses in future research to better characterize chemokine dynamics. The clinical implications of CTNNB1, TCF4, MCP1, and MCP2 expressions were further explored by correlating them with leukocyte infiltration patterns. Upregulated CTNNB1 expression was not linked to significant alterations in overall leukocyte infiltration; however, higher levels of MCP1, MCP2, or TCF4 were associated with increased infiltration of macrophages, neutrophils, and dendritic cells. In addition, enhanced TCF4 expression correlated with increased CD4+ T-cell infiltration. These findings suggest that the β-catenin/TCF4 axis may indirectly shape immune cell recruitment within the CRC TME by modulating MCP-1 and MCP-2 expression. Nonetheless, determining whether these infiltrating immune populations exert pro or anti-tumorigenic effects requires further *in vivo* characterization, which is currently underway.

## 5 Conclusion

This study demonstrates the antitumor potential of FH535. Inhibiting the Wnt/β-catenin signaling pathway directly suppresses CRC cell growth, survival, migration, and invasion, and alters chemokine expression with possible immunological effects. FH535 acts by downregulating β-catenin/TCF4-driven transcription and suppressing cyclin D1 and survivin. It induces caspase-3-mediated apoptosis and decreases MCP1 and MCP2 chemokine expression. Our in-silico results indicate that CTNNB1-dependent regulation of MCP1 and MCP2 varies by context and is likely influenced by TCF4 and other co-regulatory factors in the Wnt/β-catenin network. These results highlight the critical role of aberrant Wnt/β-catenin signaling in CRC pathogenesis and position FH535 as a promising therapeutic agent with strong antitumor activity. Its effects may be mediated by changes in chemokine expression that influence the tumor microenvironment and immune response. Further preclinical and clinical studies are required to confirm the translational potential of Wnt signaling inhibition and to clarify its immunological relevance, including its application with immunotherapeutic strategies.

## Data Availability

The original contributions presented in the study are included in the article/**Supplementary Material**. Further inquiries can be directed to the corresponding authors.

## Supplementary material

Supplementary Figure 1 Transcriptional and secretory inhibition of MCP1 and MCP2 expression by FH535 in CRC cells. Bar diagram represents quantification of **(A)** MCP1 and **(B)** MCP2 mRNA expression by Real-time quantitative PCR after RNA extraction and cDNA synthesis of FH535-treated (10, 20, and 40 µM) HCT116 and HT29 cells compared to untreated control for 24 h. Bar diagram represents quantification of secreted **(C)** MCP1 and **(D)** MCP2 protein expression in conditional media of FH535-treated (10, 20, and 40 µM) HCT116 and HT29 cells compared to untreated control for 24 h. Data are expressed as mean ± SE of a minimum of three (n=3) independent experiments, ***p < 0.001, ****p < 0.0001.

Supplementary Figure 2 THP-1 cells polarization after being treated with FH535-treated conditional media of CRC cells. Representative micrographs of polarized THP-1 cells to adherent macrophages upon FH535-treated (10, 20, and 40 µM) conditional media from **(A)** HCT116 cells and **(B)** HT29 cells for 24 h compared to untreated control. Magnification 40x, scale bar 100 µm.

Supplementary Figure 3 Quantification of cytokine markers of macrophages (M1 or M2) by ELISA assays after THP-1 cells treated with conditional media of FH535-treated CRC cells. Bar diagram represents quantification of cytokine markers **(A)** TNF-α, **(B)** IL-6, **(C)** TGF-β, and (**D**) IL-10 in conditional media of polarized macrophages from THP1 cells that were pretreated with FH-535-treated conditional media obtained from HCT116 and HT29 cells for 24 h compared to untreated control. Data are expressed as mean ± SE of a minimum of three (n=3) independent experiments, *p < 0.05, **p < 0.01, ***p < 0.001, ****p < 0.0001.
