# Supplementary material for: FH535-mediated inhibition of Wnt/β-catenin suppresses CRC growth and invasion and alters MCP1 and MCP2 expression with potential immunological implications
Source: Front Oncol. 2026 Apr 1;16:1800308. doi: 10.3389/fonc.2026.1800308 (PMC13079168; doi:10.3389/fonc.2026.1800308)
Supplement: Supplementary Figure 1 — Transcriptional and secretory inhibition of MCP1 and MCP2 expression by FH535 in CRC cells. Bar diagram represents quantification of (A) MCP1 and (B) MCP2 mRNA expression by Real-time quantitative PCR after RNA extraction and cDNA synthesis of FH535-treated (10, 20, and 40 µM) HCT116 and HT29 cells compared to untreated control for 24 h. Bar diagram represents quantification of secreted (C) MCP1 and (D) MCP2 protein expression in conditional media of FH535-treated (10, 20, and 40 µM) HCT116 and HT29 cells compared to untreated control for 24 h. Data are expressed as mean ± SE of a minimum of three (n=3) independent experiments, ***p < 0.001, ****p < 0.0001. [file SupplementaryFile1.docx]

Supplementary Figure 1


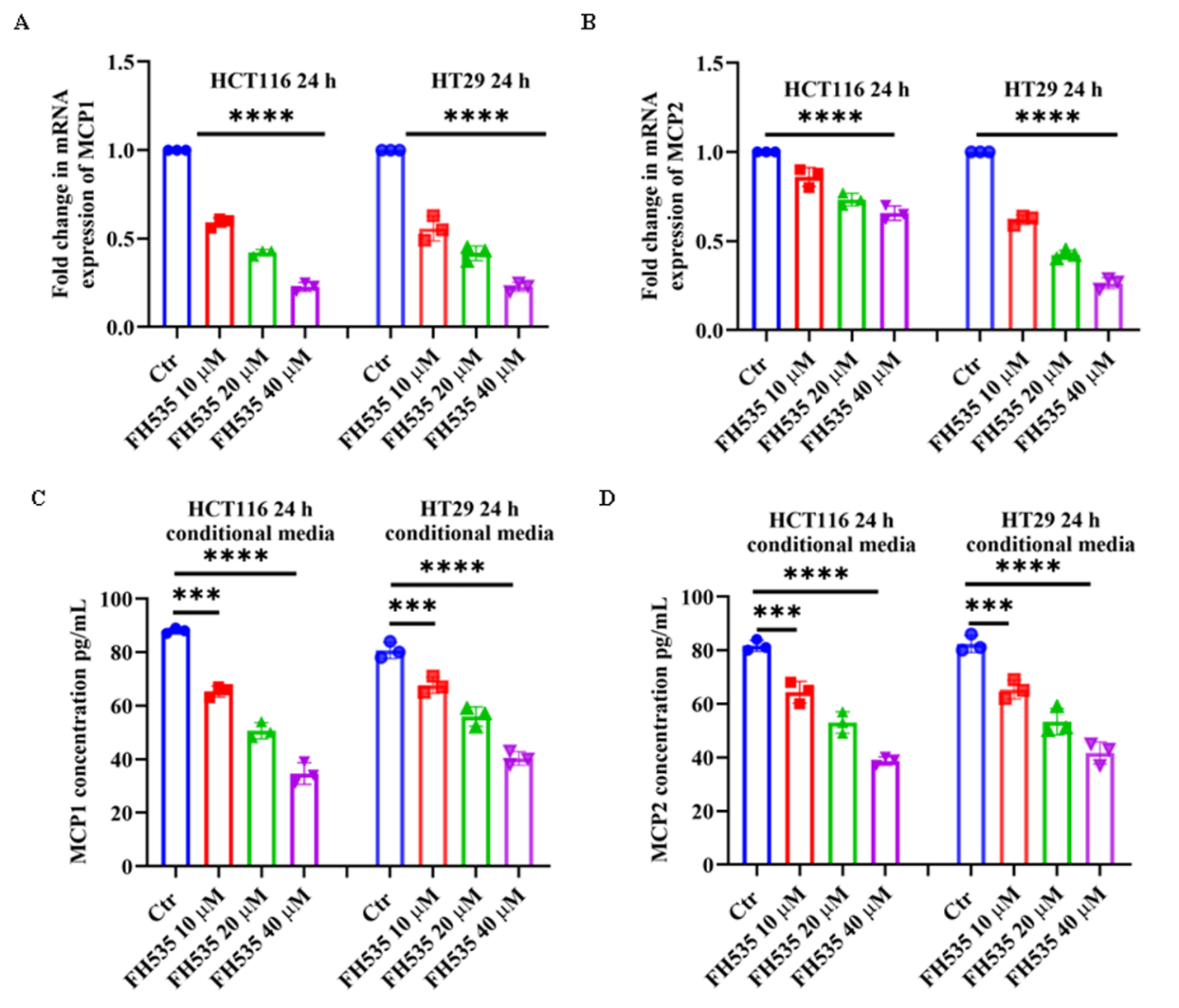


Supplementary Figure 2





Supplementary Figure 3


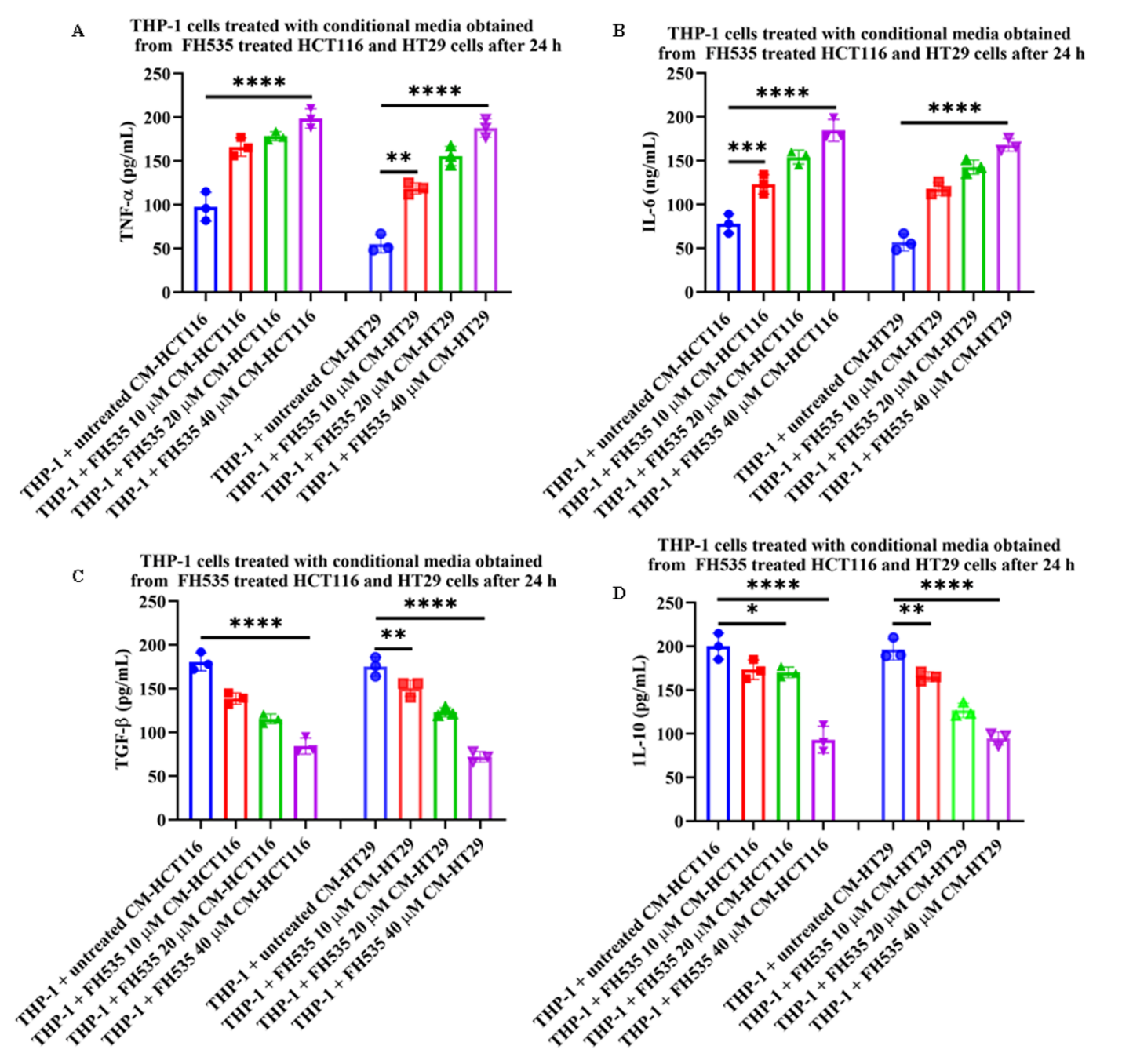


**Supplementary Figure 1.** Transcriptional and secretory inhibition of MCP1 and MCP2 expression by FH535 in CRC cells. Bar diagram represents quantification of (**A**) MCP1 and (**B**) MCP2 mRNA expression by Real-time quantitative PCR after RNA extraction and cDNA synthesis of FH535-treated (10, 20, and 40 µM) HCT116 and HT29 cells compared to untreated control for 24 h. Bar diagram represents quantification of secreted (**C**) MCP1 and (**D**) MCP2 protein expression in conditional media of FH535-treated (10, 20, and 40 µM) HCT116 and HT29 cells compared to untreated control for 24 h. Data are expressed as mean ± SE of a minimum of three (n=3) independent experiments, ***p < 0.001, ****p < 0.0001.

**Supplementary Figure 2.** THP-1 cells polarization after being treated with FH535-treated conditional media of CRC cells. Representative micrographs of polarized THP-1 cells to adherent macrophages upon FH535-treated (10, 20, and 40 µM) conditional media from (**A**) HCT116 cells and (**B**) HT29 cells for 24 h compared to untreated control. Magnification 40x, scale bar 100 µm.

**Supplementary Figure 3**. Quantification of cytokine markers of macrophages (M1 or M2) by ELISA assays after THP-1 cells treated with conditional media of FH535-treated CRC cells. Bar diagram represents quantification of cytokine markers (**A**) TNF-α, (**B**) IL-6, (**C**) TGF-β, and (**D**) IL-10 in conditional media of polarized macrophages from THP1 cells that were pretreated with FH-535-treated conditional media obtained from HCT116 and HT29 cells for 24 h compared to untreated control. Data are expressed as mean ± SE of a minimum of three (n=3) independent experiments, *p < 0.05, **p < 0.01, ***p < 0.001, ****p < 0.0001.
